# Indicators of suicidal attempt in depression

**DOI:** 10.4103/0019-5545.31572

**Published:** 2006

**Authors:** Arunima Chatterjee, Rudraprosad Chakraborty, Suprakash Chaudhary

**Affiliations:** * Junior Resident Central Institute of Psychiatry, Ranchi; ** Senior Resident RINPAS, Ranchi; *** Professor and Head RINPAS, Ranchi

Sir,

We read with interest the article by Srivastava *et al.*^1^ The authors have tried to identify the possible indicators of suicidal attempt in persons with major depressive disorder. They have examined the sociodemographic and Hamilton Depression Rating Scale (HDRS) variables among depressed persons with and without history of suicidal attempts. Such studies are necessary because cultural variations in the characteristics of persons attempting suicide have been well documented across countries.^2^ Since Srivastava *et al.*^1^ examined an outpatient group, we tried to replicate their study in an inpatient sample.

We examined 20 successive persons with a diagnosis of severe depressive episode with suicidal ideation (age= 29.80±9.41 years; 13 males) who were admitted to a psychiatric teaching hospital. None of them were suffering from any neurological, general medical or any other comorbid psychiatric illness including any substance abuse. Treating psychiatric units—blind to the aim of the study—diagnosed these patients and later reconfirmed the diagnosis in post-admission rounds according to the ICD-10 criteria. Before inclusion in the study, we obtained informed consent from all the participants. To measure depressive symptoms in the patients, we applied the HDRS–21-item version.^3^ Then we applied the Beck Scale for Suicidal Ideation (SSI)^4^ for measuring the intensity of suicidal ideation. Finally, sociodemographic and other clinical information including history of suicidal attempts during the present and past episodes were obtained. The same order of observation was maintained in all the participants to avoid any bias in rating.

A significant number of patients (*n*=10; 50%) attempted suicide in the present sample. This high frequency of suicide attempts may be due to the inpatient nature of the sample, which is expected to include more severe patients HDRS score: 26.50±4.95). The Mann–Whitney U test and chi-square test revealed no differences in sociodemographic variables such as age, sex, marital status, rural/urban background, socio-occupational status and education. Duration of present episode, number of past episodes, family history of psychiatric illness or suicide were similar. However, there were significantly more past suicidal attempts in persons who attempted suicide in the index episode (p=0.001).

An analysis of HDRS items revealed significantly more paranoid symptoms (p=0.024) in the suicide attempters and a trend towards more somatic anxiety (p=0.074) and hypo-chondriasis (p=0.075) in the non-attempters. There was no difference in the total HDRS score (p=0.268). Interestingly, Srivastava *et al.*^1^ had similar findings.

An analysis of SSI items revealed no difference in the total suicidal ideation score (p=0.414). However, persons who attempted suicide had significantly higher availability of means or opportunity for suicidal attempt (p=0.005). They also showed a trend of more specific planning (p=0.062) and deception/concealment of the contemplated attempt (p=0.081).

Since our study population was small, there is always a chance of error associated with small samples. However, our effort probably indicates the presence of some genuine indicators of an imminent suicide attempt in a depressed person. A well-designed study is therefore urgently needed in this area.
